# Changes in cerebral glucose metabolism among mild long COVID patients: an [18F]FDG PET/CT study

**DOI:** 10.1590/1414-431X2024e14228

**Published:** 2024-11-25

**Authors:** J.S. Sakamoto, L.E. Lopes-Santos, K.J.C.C. de Lacerda, A.C. Trevisan, L. Alexandre-Santos, O.Y. Fukumori, F. Bellissimo-Rodrigues, L. Wichert-Ana

**Affiliations:** 1 Laboratório de Medicina Nuclear e PET/C, Departamento de Imagem Médica, Hematologia e Oncologia Clínica, Faculdade de Medicina de Ribeirão Preto, Universidade de São Paulo, Ribeirão Preto, SP, Brasil; 2 Departamento de Medicina Social, Faculdade de Medicina de Ribeirão Preto, Universidade de São Paulo, Ribeirão Preto, SP, Brasil

**Keywords:** COVID-19, Long COVID, Glucose metabolism, Brain PET/CT

## Abstract

COVID-19, caused by SARS-CoV-2, presents diverse symptoms, including neurological manifestations. This study investigated COVID-19's neurological sequelae, focusing on the central nervous system's involvement through cerebral glycolytic metabolism assessed via PET/CT. Twenty-two patients with mild long COVID cognitive symptoms and 20 healthy volunteers without cognitive, psychiatric, or neurological impairments and no history of COVID-19 infection underwent cerebral PET/CT scans using [18F]FDG to assess cerebral metabolism. The study meticulously evaluated the uptake of [18F]FDG in various brain regions, employing the CortexID Suite software for quantitative analysis. The analysis focused on identifying areas of hypometabolism and hypermetabolism, indicative of altered glucose metabolism possibly related to COVID-19's neurological impact. No statistically significant differences were found between the mild COVID and healthy groups. Although our sample was too small to generate a statistical difference between groups, future studies should explore some findings, such as hypometabolism in 15 regions and hypermetabolism in 11 regions in the mild COVID group. These changes, especially in areas linked to executive functions, sensory perception, and emotional regulation, suggest nuanced alterations in brain function. Our study did not find significant glycolytic metabolic changes in patients with mild long COVID. However, areas of glycolytic hypometabolism and hypermetabolism found in some patients showed biological plausibility with the cognitive and affective symptoms they presented. Future investigations with a larger sample size should be correlated with neuropsychological and neuropsychiatric examinations to confirm this relationship.

## Introduction

The 2019 coronavirus disease (COVID-19), caused by the severe acute respiratory syndrome coronavirus 2 (SARS-CoV-2), is a highly contagious and harmful respiratory illness. The virus requires the essential receptor angiotensin-converting enzyme 2 (ACE2) to enter the cell and start its replication process (1). Severe disease is associated with a highly dysregulated innate immune response, characterized by a delayed response to interferon (IFN), essential for immune defense against microbial pathogens, resulting in an exaggerated inflammatory response and consequently increased organ tissue damage (2,3). While most COVID-19 patients primarily develop respiratory symptoms, there has been an increase in neurological symptoms and manifestations associated with the disease (4).

Although ACE2 levels are lower in the brain, specific brain regions express relatively high levels (5), leading to neurological symptoms that can occur independently or in conjunction with respiratory symptoms, potentially affecting three different systems: the central nervous system (CNS), the peripheral nervous system (PNS), and the musculoskeletal system (6,7). Neurological complications of COVID-19, such as encephalopathy, encephalitis, and Guillain-Barré syndrome (GBSs) (8-10), may persist after recovery from acute infection, including cognitive impairments and prolonged symptoms such as headache, mental confusion, disturbed sleep, anosmia, fatigue, post-exertional malaise, anxiety, and post-traumatic stress (11-13).

Positron emission tomography (PET) with [18F]FDG has been used to investigate brain metabolic changes in post-COVID-19 patients, revealing hypometabolism in various brain regions (14). Studies on cerebral PET/CT in COVID-19 have been widely performed and refined to identify areas of increased inflammation and infection due to COVID-19 and cerebral metabolic dysfunction. Some studies have shown that post-COVID-19 patients exhibited hypometabolism in several brain regions, primarily in the orbitofrontal cortex, olfactory gyrus, right temporal lobe, and frontoparietal areas (15-17). Thus, brain PET/CT with [18F]FDG can help investigate neurobiological structures possibly affected either by the direct action of the Sars-Cov-2 virus or by the cerebral inflammatory response to the infection.

In this study, we examined 22 patients with non-hospitalized post-COVID-19 cognitive complaints and 20 Brazilian volunteers to identify changes in brain metabolism. This approach aimed to enhance our understanding of the neurological sequelae of COVID-19 and facilitate the development of diagnostic and therapeutic strategies for patients affected by these complications.

## Material and Methods

### Study design

The participants were divided into a control group and a mild COVID-19 group. The recruitment included 22 patients aged between 18 and 60 years, infected for at least two months and no more than 12 months at the time of recruitment, not hospitalized, with cognitive complaints after COVID-19, not using psychiatric medication, without a history of psychiatric or neurological disorders, without diabetes or hypertension, and with at least complete high school.

Additionally, a control group of 20 individuals was recruited (aged between 18 and 60 years), who had no COVID-19 symptoms in the last two years or tested negative for COVID-19 during the pandemic, without cognitive complaints, not using psychiatric medication, without a history of psychiatric or neurological disorders, without a previous diagnosis of neurodevelopmental disorders, and with at least complete high school.

### Subjects

The research material consisted of PET/CT images with FDG-18 from 42 individuals, both female (73.8%) and male (26.2%), comprising 22 patients post SARS-CoV-2 with mild symptoms and 20 individuals without any cognitive, psychiatric, or neurological impairments, obtained from the database of the Hospital das Clínicas da Faculdade de Medicina de Ribeirão Preto (HC-FMRP-USP). The hospital's Research Ethics Committee approved the study under protocol number (CAAE: 56334022.5.0000.5440). All participants were informed of the study's procedures and conditions for participation and agreed to sign the informed consent form.

### Neuropsychological assessment

The psychology team individually conducted a comprehensive neuropsychological assessment with each participant at HC-FMRP-USP. This assessment took approximately 4 h, with 10-min breaks every hour to ensure optimal concentration. The environment was carefully selected to be free of distractions. The assessment began with an interview to confirm the participant's medical history and gather information about their cognitive complaints. A battery of standardized tests was administered to evaluate various cognitive functions. These included assessments of intelligence using the Wechsler Abbreviated Scale of Intelligence (WASI), attention, and executive functions using tests such as the d2-R, Trail Making Test, Stroop Test, Digit Span, Verbal Fluency, and Wisconsin Card Sorting Test (WCST). Verbal memory was assessed using the Logical Memory and Rey Auditory Verbal Learning Test (RAVLT), while visual memory was evaluated using the Visual Reproduction and Rey Complex Figures tests. Additionally, language abilities were assessed using the Boston Naming Test. The protocol was standardized and systematically administered to all participants.

### [18F]FDG PET/CT Protocol

#### Patient preparation

The patient was instructed to fast for at least 4 hours, except for water. The radiopharmaceutical [18F]FDG was administered via venous access. Blood pressure was within normal range, and blood glucose levels were up to 200 mg/dL. Volunteers were awake and with their eyes open, and the room was dimly lit, without visual or auditory stimuli.

#### Radiopharmaceutical fractionation and administration

The dose calibrator underwent daily verification and quality control. The individual dose of [18F]FDG was fractionated into doses of 10 mCi at 2.5 to 3.0 mL per syringe, supplemented, if necessary, with saline solution. The [18F]FDG dose was stored in specific shielding until the nursing team administered the injection after 30 min following the venous puncture. After [18F]FDG administration, volunteers remained at rest and free from stimuli for one hour, the time necessary for the biodistribution of the radiopharmaceutical in the cerebral and cerebellar parenchyma.

#### Acquisition and processing of cerebral PET/CT

Images were acquired using a GE Discovery MI PET/CT scanner (GE Healthcare, USA). The PET scanner has four rings of LYSO (lutetium-yttrium oxyorthosilicate) crystals combined with a 64-channel physical CT. The total acquisition time was 15 minutes. To determine rCMRglu, the maximum standardized uptake value (SUVmax) rather than the mean SUV was quantified in the brain, whether within the pixel, voxel, or cluster (18).

### Processing and analysis of cerebral PET/CT images using CortexID suite

For the analysis of cerebral metabolism of [18F]FDG, SUVs were obtained for each volume of interest (VOI) and normalized to the global distribution of the radiopharmaceutical to get a SUV ratio (SUVr) for each [18F]FDG PET. We avoided using the pons and cerebellum as reference regions, which resulted in higher data heterogeneity, potentially leading to an underestimation of the SUVr of different VOIs. Therefore, this study proposed the global region as the reference region for calculating the SUVr of different VOIs.

The PET/CT images were interpreted through quantitative analysis using the CortexID Suite software (GE Healthcare). This software quantitatively assesses each subject's cerebral [18F]FDG metabolism compared to a brain PET database of healthy volunteers installed on the PET/CT workstation. The metabolic rates of individual patient VOIs were described by the Z-scores generated in CortexID for the following 26 regions: right and left lateral prefrontal, right and left medial prefrontal, right and left sensorimotor, right and left anterior cingulate, right and left posterior cingulate, right and left precuneus, right and left superior parietal, right and left inferior parietal, right and left lateral occipital, right and left primary visual, right and left lateral temporal, right and left mesial temporal, cerebellum, and pons.

**Figure 1:**
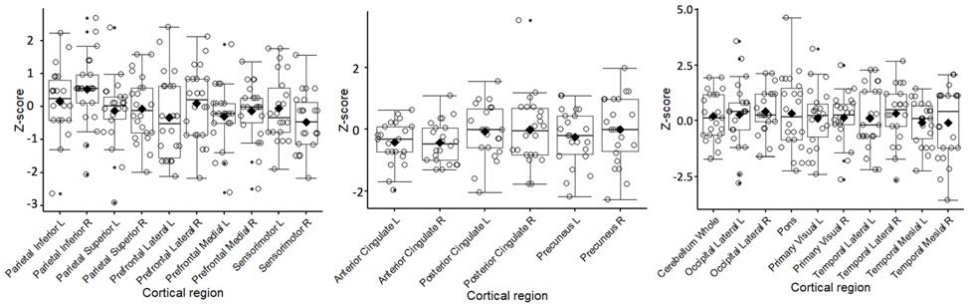
Boxplot of the glycolytic metabolism Z-score by cortical regions in 22 patients with mild COVID. The values represent each cortical region's median (horizontal black bar) and mean (black rhombus).

We considered glycolytic hypometabolism to be significant if the VOI's Z-score was lower than -2 (below the threshold of two standard deviations for each cortical region) and hypermetabolism if the VOI's Z-score was higher than +2 (above the threshold of two standard deviations). The dispersion of the mean VOI's Z-scores for the group of 22 mild long COVID is shown in Figure 1. Boxplots depict the minimum and maximum values and the frequency of a Z-score less than -2 and more than +2 for each VOI. The values are described in Table 1.

**Table 1:** Comparison of standardized uptake values (SUVs) for different cortical regions between mild long COVID and healthy control groups.

Cortical region	Mild COVID (n=22)mean (SD)	Healthy group (n=20) mean (SD)	Student's *t*-test P-value*	Mean Z-score**
R Prefrontal Lateral	0.9927 (±0.0254)	0.9905 (±0.0232)	0.7687	0.0956
L Prefrontal Lateral	1.0090 (±0.0284)	1.0165 (±0.0220)	0.3489	-0.3357
R Prefrontal Medial	0.9454 (±0.0323)	0.9505 (±0.0363)	0.6384	-0.1388
L Prefrontal Medial	0.9568 (±0.0319)	0.9670 (±0.0334)	0.3202	-0.3046
R Sensorimotor	0.9100 (±0.0261)	0.9240 (±0.0294)	0.1127	-0.4753
L Sensorimotor	0.9554 (±0.0359)	0.9575 (±0.0355)	0.8539	-0.0575
R Anterior Cingulate	0.8781 (±0.0447)	0.9070 (±0.0670)	0.1128	-0.4297
L Anterior Cingulate	0.8831 (±0.0499)	0.9140 (±0.0731)	0.1223	-0.4212
R Posterior Cingulate	1.1222 (±0.0746)	1.1235 (±0.0640)	0.9546	-0.0191
L Posterior Cingulate	1.1259 (±0.0613)	1.1315 (±0.0696)	0.7848	-0.0802
R Precuneus	1.0704 (±0.0436)	1.0710 (±0.0399)	0.9665	-0.0136
L Precuneus	1.0581 (±0.0413)	1.0700 (±0.0459)	0.3883	-0.2569
R Parietal Superior	0.9031 (±0.0584)	0.9070 (±0.0589)	0.8343	-0.0647
L Parietal Superior	0.8972 (±0.0584)	0.9040 (±0.0565)	0.7067	-0.1190
R Parietal Inferior	0.9295 (±0.0249)	0.9175 (±0.0231)	0.1126	0.5205
L Parietal Inferior	0.9431 (±0.0237)	0.9395 (±0.0225)	0.6098	0.1629
R Occipital Lateral	1.0154 (±0.0317)	1.0015 (±0.0323)	0.1664	0.4316
L Occipital Lateral	1.0068 (±0.0375)	1.0000 (±0.0251)	0.4901	0.2713
R Primary Visual	1.2190 (±0.1047)	1.2060 (±0.0937)	0.6714	0.1396
L Primary Visual	1.2468 (±0.1078)	1.2370 (±0.0780)	0.7356	0.1258
R Temporal Lateral	0.8018 (±0.0266)	0.7950 (±0.0206)	0.3574	0.3302
L Temporal Lateral	0.8163 (±0.0283)	0.8140 (±0.0201)	0.7555	0.1175
R Temporal Mesial	0.6440 (±0.0348)	0.6460 (±0.0213)	0.8301	-0.0893
L Temporal Mesial	0.6545 (±0.0292)	0.6570 (±0.0301)	0.7904	-0.0815
Cerebellum Whole	0.7027 (±0.0413)	0.6950 (±0.0384)	0.5337	0.2009
Pons	0.5845 (±0.0491)	0.5750 (±0.0291)	0.4436	0.3279

*P<0.05 is considered statistically significant. **Negative values indicate hypometabolic regions and positive values indicate hypermetabolic regions. L: left; R: right.

The software (CortexID) was certainly not adapted for scientific purposes, mainly because the database of healthy volunteers is not transparent regarding demographic variables. To overcome this limitation, we used a resource that allows us to extract more reliable comparative data to perform two analyses. The first was to compare the brain PET of each of the 20 healthy volunteers of our study with the CortexID healthy database and obtain the Z-score dispersion from each brain area. The second analysis compared the Z-score dispersion of each of the 22 patients with mild long COVID with the same database. Finally, to compare the data dispersion of the mild long COVID group and the healthy group, a new Z-score was calculated based on the SUV and standard deviation of the healthy control group using the following equation:

$${\bar{Z}}_{\text{score}}=\frac{1}{n}\sum_{i=1}^{n}\left(SUV_{i}-{\bar{SUV}}_{control}/\sigma_{SUV_{\text{control}}}\right)$$ (Eq. 1)

### Statistical analysis

A comprehensive statistical analysis was conducted using descriptive and inferential techniques. Descriptive analysis was employed to summarize data measures, such as means, standard deviations, and confidence intervals. The inferential analysis assessed whether differences between sample means were statistically significant. The Student's *t*-test was used for this assessment. The Statistical Package Social Sciences (SPSS, version 25.0, IBM Corporation, USA) was to used for Fisher's exact tests for simple statistical comparison of differences in the number of vaccine doses between long COVID and healthy control groups. In all analyses, a P-value <0.05 was considered statistically significant.

## Results

This study compared images of the brain [18F]FDG PET/CT of 22 patients with long COVID, four men and 18 women with an average age of 38.77 (SD 1.46) years with a native database of PET/CT from 20 healthy volunteers within the same age range who had no prior history of COVID-19 (7 men and 13 women with an average age of 40.65 years). The demographic and clinical characteristics are summarized in Table 2 and detailed in Table 3. The mean interval between the onset of COVID-19 infection and the onset of cognitive symptoms was 56 (SD 46.75) days, and the mean delay for the start of clinical investigation of the long COVID was 208 (SD 57.31) days.

**Table 2:** Demographic characteristics of patients with mild COVID-19 and healthy controls.

	Mild COVID group	Healthy group
Patients (n)	22	20
Age (years)	38.77±11.47	40.65±8.15
Gender (male/female)	4/18	7/13
Cognitive complaints		
Slow thinking	81.82%	-
Lack of concentration	81.82%	-
Memory loss	100.00%	-
Mood change	18.18%	-
Left/right-handed (%)	0/100	5/95
Vaccine doses* (%)		
1	4.55%	00.00%
2	13.64%	00.00%
3	72.73%	00.00%
4	9.10%	80.00%
PCR time	216.5±63.77	-

Data are reported as means and SD or number and percentage. *P<0.001, Fisher exact test.

**Table 3:** Demographic, clinical, and imaging data of individual patients.

Pt	Gender	Age (yr)	Vaccines (n of doses)	Days with cognitive symptoms	Days before evaluation	Cognitive symptoms	PET/CT findings
1	F	40	3	30	203	Forgetfulness of routine things, names of people, and slow reasoning	Normal
2	F	54	3	90	178	Forgetfulness of names, slow reasoning, difficulty organizing ideas	Normal
3	F	52	3	30	238	Forgetfulness of words, slow reasoning, lack of concentration	(+) posterior cingulate, precuneus parietal(-) R parietal, Bi mesial temporal
4	F	44	3	60	145	Forgetfulness of tasks and names, slow reasoning, lack of concentration	Normal
5	F	55	3	60	273	Forgetfulness of names, slow reasoning, lack of concentration	(-) Bi anterior temporal
6	F	52	3	90	150	Forgetfulness of names, slow reasoning, lack of concentration	(+) posterior cingulate
7	F	30	3	90	126	Forgetfulness of names, slow reasoning, lack of concentration	(+) posterior cingulate, precuneus parietal.
8	F	33	3	60	277	Forgetfulness of words, slow reasoning, lack of concentration	(+) posterior cingulate.(-) Bi mesial temporal
9	F	33	4	14	134	Forgetfulness of names and new information, slow reasoning, lack of concentration	(+) posterior cingulate
10	M	37	4	30	174	Forgetfulness of names and tasks, slow reasoning, lack of concentration	(+) posterior cingulate, (-) Bi anterior mesial temporal, Bi insula
11	M	33	3	30	260	Forgetfulness of names, slow reasoning, lack of concentration, mood swings	Normal
12	M	49	3	60	140	Forgetfulness of tasks, slow reasoning, lack of concentration, mood swings	Normal
13	F	27	3	21	221	Forgetfulness of words, slow reasoning	(+) posterior cingulate(-) Bi temporal
14	F	47	2	180	222	Forgetfulness of names and words, slow reasoning, mood swings	(+) posterior cingulate
15	F	30	3	14	245	Sluggishness, slow reasoning, forgetfulness of tasks and names	(+) posterior cingulate, precuneus parietal
16	F	57	3	21	217	Disposition, irritability, fatigue, forgetfulness of dates, slow reasoning, lack of concentration	Normal
17	F	49	3	30	216	Forgetfulness of recent facts, slow reasoning, distraction	Normal
18	F	25	3	30	130	Forgetfulness of names and words, slow reasoning	Normal
19	F	30	1	120	289	Forgetfulness of names and tasks, slow reasoning	Normal
20	F	24	3	90	165	Forgetfulness of tasks, slow reasoning	(+) posterior cingulate
21	M	32	2	30	293	Forgetfulness of recent facts, lack of concentration	(+) posterior cingulate
22	F	20	2	60	289	Forgetfulness of names and words, lack of concentration	Normal
Mean		38.77		56.00	208.00		
SD		1.46		40.75	57.31		

Pt: patient; yr: years; F: female; M: male; m: mean; SD: standard deviation.

### [18F]FDG PET/CT findings

Table 3 describes the visual analysis of the brain [18F]FDG PET/CT of 22 mild long COVID patients.

The level of brain metabolism was expressed as a Z-score value, which indicates how much a measurement deviates from the mean in terms of standard deviation. In studies of [18F]FDG, negative Z-score values indicate glucose hypometabolism and positive values indicate hypermetabolism. There was no statistically significant difference across all regions suggesting mild COVID did not cause brain damage (Table 1). As all P-values were >0.05, no adjustment method, such as the Bonferroni correction, was used. However, when analyzing the average Z-score normalized by the control group, we found 15 regions with [18F]FDG hypometabolism and 11 brain regions with hypermetabolism. Patients with mild COVID exhibited glycolytic hypometabolism in the left prefrontal medial and lateral areas and the bilateral projections of the prefrontal medial, sensorimotor, anterior and posterior cingulate, precuneus, superior parietal, and mesial temporal regions. The same mild COVID group also showed glycolytic hypermetabolism in the right lateral prefrontal regions and bilateral projections of the inferior parietal, mesial, lateral occipital, lateral temporal areas, cerebellum, and pons.

**Figure 2:**
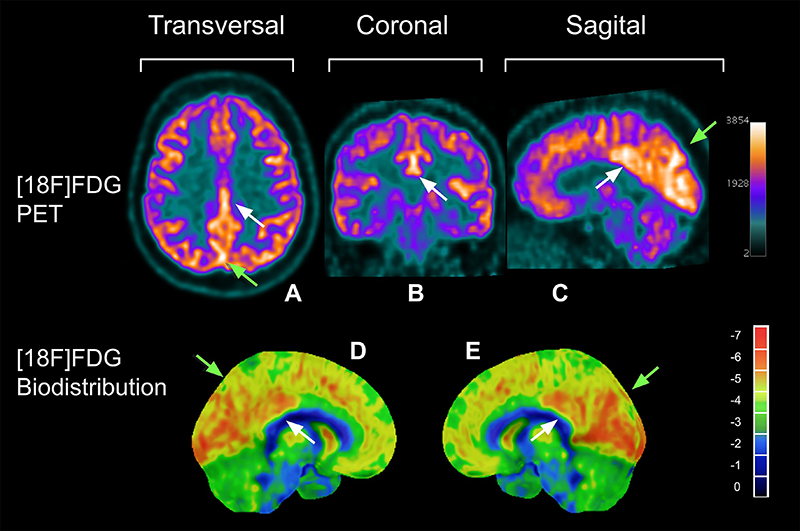
**A**, **B**, and **C**, transversal, coronal, and sagittal brain views by [18F]FDG PET of a patient with glycolytic hypermetabolism. **D** and **E**, bilateral mesial aspects of a 3D exhibition of the biodistribution of the radiopharmaceutical on the brain via CortexID^©^ software (General Electric). White arrows indicate the cerebral cortex with glycolytic hypermetabolism on the posterior cingulate, and green arrows indicate the precuneus parietal regions. FDG: fluorodeoxyglucose; PET: positron emission tomography.

**Figure 3:**
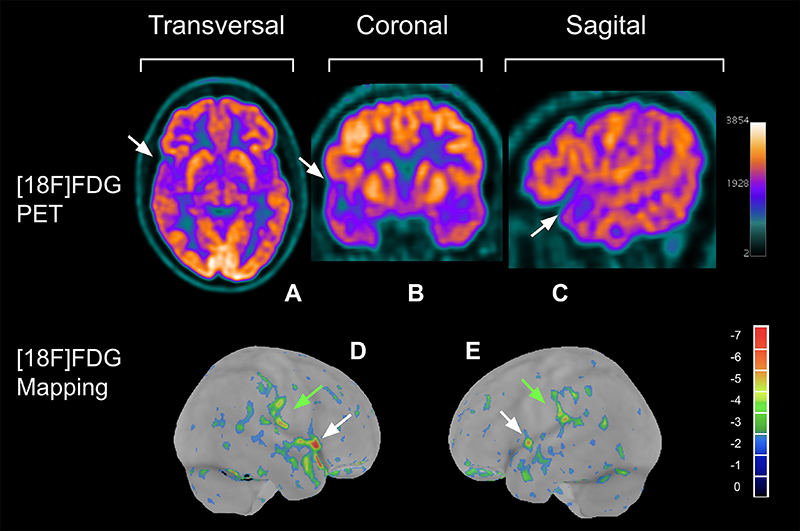
**A**, **B**, and **C**, transversal, coronal, and sagittal brain views by [18F]FDG PET of a patient that presents glycolytic hypometabolism. **D** and **E**, bilateral mesial aspects of a 3D statistical brain mapping of the radiopharmaceutical uptake compared to a healthy subject database via CortexID^©^ software (General Electric). White arrows indicate the cerebral cortex with glycolytic hypometabolism on the lateral and anterior aspects of the temporal lobes, and green arrows indicate the hypometabolism on the inferior lobules of the parietal lobes. FDG: fluorodeoxyglucose; PET: positron emission tomography.

Table 4 demonstrates a significant variation in the minimum and maximum Z-score values (min; max) for each cerebral VOI of patients with mild long COVID. Significant hypometabolism (Z-score ≤ -2) and hypermetabolism (Z-score ≥ +2) were observed in several cortical regions, albeit in a small number of patients. Some VOIs exhibited both hypometabolism and hypermetabolism among the 22 patients, with three VOIs standing out: left lateral occipital and left lateral temporal regions (two patients presented hypometabolism and two others, hypermetabolism), and right mesial temporal region (three patients presented hypometabolism and two, hypermetabolism), which showed a relatively higher number of patients with significant findings, in a small fraction of patients (4.55% in each case). Notably, the right mesial temporal region evidenced the highest prevalence of significant hypometabolism, affecting 13.64% of patients. Additionally, considerable hypermetabolism was observed in the pons and left lateral occipital and lateral temporal regions, affecting 9.09% of patients. Five cerebral VOIs were spared of significant hypo- or hypermetabolism: the left sensory, right anterior cingulate, left anterior cingulate, right superior parietal, and whole cerebellum regions. Figures 2 and 3 show patients with glycolytic hyper- and hypometabolism, respectively.

**Table 4:** Z-score values (minimum and maximum) for each cerebral volume of interest (VOI) in the group with mild long COVID.

Cortical region	min; max	Z-score [≤ -2; n (%)]**	Z-score [≥ +2; n (%)]**
R Prefrontal Lateral	-2.1694; +2.1265	1 (4.55%)	1 (4.55%)
L Prefrontal Lateral	-2.1068; +2.4240	1 (4.55%)	1 (4.55%)
R Prefrontal Medial	-2.4901; +1.3620	1 (4.55%)	0
L Prefrontal Medial	-2.6032; +1.8851	1 (4.55%)	0
R Sensorimotor	-2.1730; +1.5619	1 (4.55%)	0
L Sensorimotor	-1.9002; +1.7594	0	0
R Anterior Cingulate	-1.2975; +1.0887	0	0
L Anterior Cingulate	-1.9684; +0.6288	0	0
R Posterior Cingulate	-1.7730; +3.5383	0	1 (4.55%)
L Posterior Cingulate	-2.0306; +1.5570	1 (4.55%)	0
R Precuneus	-2.2757; +1.9756	1 (4.55%)	0
L Precuneus	-2.1740; +1.0870	1 (4.55%)	0
R Parietal Superior	-1.9848; +1.5776	0 (0%)	0
L Parietal Superior	-2.9020; +2.4065	1 (4.55%)	1 (4.55%)
R Parietal Inferior	-2.0525; +2.7007	1 (4.55%)	2 (9.09%)
L Parietal Inferior	-2.6340; +2.2356	1 (4.55%)	1 (4.55%)
R Occipital Lateral	-1.5931; +2.1190	0	2 (9.09%)
L Occipital Lateral	-2.7853; +3.5812	2 (9.09%)	2 (9.09%)
R Primary Visual	-2.6248; +2.4968	1 (4.55%)	1 (4.55%)
L Primary Visual	-2.3971; +3.2432	1 (4.55%)	2 (9.09%)
R Temporal Lateral	-2.6637; +2.6637	1 (4.55%)	1 (4.55%)
L Temporal Lateral	-2.1885; +2.2879	2 (9.09%)	2 (9.09%)
R Temporal Mesial	-3.5557; +2.0585	3 (13.64%)	2 (9.09%)
L Temporal Mesial	-2.2255; +1.4283	1 (4.55%)	0
Cerebellum Whole	-1.6901; +1.9502	0	0
Pons	-2.2329; +4.6376	1 (4.55%)	2 (9.09%)

min; max: minimum and maximum values of the Z-score found in a specific volume of interest (VOI) within the group of 22 patients with mild long COVID. **Number of patients with mild COVID with significant hypometabolism (Z-score ≤2) or hypermetabolism (Z-score ≥2) within each VOI. L: left; R: right.

These findings suggested that hypometabolism and hypermetabolism in specific brain regions may be characteristics of mild long COVID-19, possibly reflecting variations in the brain's response to the virus. A detailed analysis of these metabolic alterations may offer valuable insights into the underlying mechanisms of mild long COVID-19 and potentially guide future therapeutic strategies.

## Discussion

This [18F]FDG cerebral PET/CT study identified alterations in cerebral metabolism in patients with mild long COVID compared to healthy individuals. A small percentage of patients were found to have significant hypometabolism or hypermetabolism in different brain cortical regions. The highest rate of cerebral hypometabolism (13.64% of patients) was found in the right mesial temporal region, which plays an essential role in memory formation and retrieval. On the other hand, the highest rate of significant cerebral hypermetabolism alterations was found in seven different cortical regions: right inferior parietal, right and left lateral occipital, left primary visual, left lateral temporal, right mesial temporal, and pons regions; however, fewer patients were affected, with only 9.09% of patients having the alteration in each cerebral region, which were related to emotion processing, sensory integration, and visual information.

The percentages of individuals with significant alterations in cerebral metabolism were small. Despite patients reporting impairments in work and daily life due to neurocognitive symptoms, quantitative analyses performed using CortexID software did not produce regions with significantly different glucose metabolism in patients with mild long COVID compared to control patients.

These results indicated that patients with mild long COVID-19 may present discrete neurocognitive impairments. Patients with long COVID presented impaired executive functions as well as affected attention, memory, voluntary movement control, and sensory perception, which may be related to the mild hypometabolism observed in the prefrontal, sensorimotor, anterior and posterior cingulate, precuneus, superior parietal, and mesial temporal regions. On the other hand, patients also showed impaired emotional control, as well as compromised sensory integration and visual information, which may be related to the mild hypermetabolism observed in the right lateral prefrontal, inferior parietal, lateral occipital, primary visual, lateral temporal, cerebellum, and pons regions.

These findings are in line with other studies. A study by Voruz et al. (19) reported deficits in memory, attention, executive function, and mood alteration in patients with mild disease. Zhao et al. (20) also reported that asymptomatic to moderate COVID-19 survivors exhibited worsening episodic memory and decreased attention and task execution time.

The findings of our study, which used cerebral [18F]FDG PET/CT to assess glycolytic metabolism alterations associated with long COVID, agreed with reports by Dressing et al. (21), where patients did not show significant hyper- or hypometabolism. A study by Hugon et al. (22) also showed mild hypometabolism in the left parietal, precuneus, and anterior and posterior cingulate cortex regions.

Regarding the limitations of the present study, only patients with mild long COVID who were infected with SARS-CoV-2 and presented mild symptoms were included in our cohort, representing a small subgroup of COVID-19 patients. Additionally, the CortexID software compares data with an American database, which limits comparisons with our Brazilian control group. This issue was addressed by calculating the standardized Z-score. However, the results are similar to those of other studies. Lastly, future research is needed to provide more robust support and further analysis, and additional studies are required to fully understand the implications of these metabolic differences and their clinical significance, as well as the impact of metabolic alterations at other levels of disease severity.

### Conclusion

Our study did not find significant glycolytic metabolic changes in patients with mild long COVID. However, our results showed discrete cerebral glycolytic metabolism findings, manifesting as hypometabolism and hypermetabolism, among patients experiencing mild long COVID symptoms. Future research should correlate cerebral glycolytic metabolism rates with emotional, affective, and neuropsychological evaluation scores, enhancing our understanding of the long-term neurocognitive impact of COVID-19.
